# Detection of sentence boundaries and abbreviations in clinical narratives

**DOI:** 10.1186/1472-6947-15-S2-S4

**Published:** 2015-06-15

**Authors:** Markus Kreuzthaler, Stefan Schulz

**Affiliations:** 1 Institute for Medical Informatics, Statistics and Documentation, Medical University of Graz, Auenbruggerplatz 2, 8036 Graz, Austria

**Keywords:** clinical narrative, natural language processing, machine learning

## Abstract

**Background:**

In Western languages the period character is highly ambiguous, due to its double role as sentence delimiter and abbreviation marker. This is particularly relevant in clinical free-texts characterized by numerous anomalies in spelling, punctuation, vocabulary and with a high frequency of short forms.

**Methods:**

The problem is addressed by two binary classifiers for abbreviation and sentence detection. A support vector machine exploiting a linear kernel is trained on different combinations of feature sets for each classification task. Feature relevance ranking is applied to investigate which features are important for the particular task. The methods are applied to German language texts from a medical record system, authored by specialized physicians.

**Results:**

Two collections of 3,024 text snippets were annotated regarding the role of period characters for training and testing. Cohen's kappa resulted in 0.98. For abbreviation and sentence boundary detection we can report an unweighted micro-averaged F-measure using a 10-fold cross validation of 0.97 for the training set. For test set based evaluation we obtained an unweighted micro-averaged F-measure of 0.95 for abbreviation detection and 0.94 for sentence delineation. Language-dependent resources and rules were found to have less impact on abbreviation detection than on sentence delineation.

**Conclusions:**

Sentence detection is an important task, which should be performed at the beginning of a text processing pipeline. For the text genre under scrutiny we showed that support vector machines exploiting a linear kernel produce state of the art results for sentence boundary detection. The results are comparable with other sentence boundary detection methods applied to English clinical texts. We identified abbreviation detection as a supportive task for sentence delineation.

## Introduction

The full stop or period character is known to be ambiguous. Besides its primary use as a sentence delimiter, the period is often collocated with an abbreviation ("e.g.", "etc.", "Prof."). Periods also occur in numeric expressions ("13.2 mg") including dates ("24.1.2014"), as well as in a series of specialized names like file names ("readme.txt"), web addresses ("http://www.wikipedia.org"), or codes (e.g. International Classification of Diseases: "A01.9"). This is roughly true for all Western languages; yet minor variations exist between languages and dialects, e.g., the use of the period as decimal delimiter, its use in date and time formats, or the rules that guide its collocation with abbreviations.

A character-wise analysis of text allows for a distinction between period characters that are enclosed between two alphanumeric characters, and period characters that are followed by at least one, non-alphabetic character, such as a further punctuation sign, a space, tab or new line. The latter phenomenon is the focus of this study. Three cases are distinguished:

• Period characters that constitute the last character of an abbreviation.

• Period characters that follow non-abbreviated words and act as sentence delimiters.

• Period characters that are part of an abbreviation and additionally delimit a sentence.

In cases where the period is enclosed between two alphanumeric characters, it is considered an internal part of a token. This may be confounded with cases in which the space after a period is erroneously omitted, which masks token and sentence boundaries. However, we consider the correction of such punctuation errors as a separate problem outside the scope of this study.

If the roles of period characters are not appropriately clarified, sentences are split improperly, which has a severe impact on text analytics. In addition, if a system fails to identify abbreviations, their interpretation by mapping to full forms is impaired. Compared to fictional or journalistic texts, this distinction is particularly relevant for narrative clinical notes, in which periods are much more frequent [1]. We will investigate methods for identifying and classifying period characters in these text types, as a sub task of the so-called sentence boundary detection problem.

Our clinical documentation use case focuses on text as it is typed into the computer by the physician at the point of care, or alternatively dictated and then processed by professional typists. In general, narratives constitute the most comprehensive and elaborate part of electronic patient records. Discharge summaries, in particular, constitute a rich abstract of those facts in a treatment episode that are considered relevant for decision making. Thus, discharge summaries are important vehicles for inter-physician communication, but they have also been increasingly valued as a rich source for the extraction of clinical information within so-called secondary use scenarios [2].

Clinical language is characterized, among other peculiarities like misspellings, punctuation errors and incomplete sentences, by an abundance of acronyms and abbreviations [3]. This is why we focus here on the use of the period character to distinguish between sentence limits and abbreviations. Our texts are in the German language, where abbreviations are nearly mandatorily followed by a period - in contrast to acronyms, which catch one's eye by the collocation of several capital letters and, occasionally, digits. Non acronymic non-period abbreviations (like "Prof") are generally disallowed. Physicians comply surprisingly well with this rule (in contrast to other editing errors they commit), and the exceptions are limited to a few frequent and short examples (e.g. "li", "supp", "caps").

We have also observed a tendency towards unusually lengthy abbreviations, chosen as a means to abbreviate long words (often single-word compounds) at the point where their (visual) completion by the reader can be taken for granted. Examples: "Penicillinallerg." ("...ie"), "melanozyt." ("...ische"), "paraffineingebett." ("...et"). As long as they are intuitively understandable, these ad-hoc abbreviations are tolerated in medical notes, although they would never be admitted by medical publishers. Ad-hoc abbreviations are not lexicalized, but they commonly constitute substrings that are specific to a lexicon entry (albeit not necessarily to any determined inflection or derivation).

Periods also frequently occur as markers for ordinal numbers, where we observe similar ambiguities. A snippet like "5." may be read as a cardinal number followed by a sentence delimiter in "The first convulsion occurred at the age of 5.". In "it was the 5. occurrence" it must be read as a ordinal number, at least in German, in which the period is a mandatory marker for ordinals, in default of special markers like 5*^th^*, 5*^o ^*or 5*^ème ^*. Finally, in "This fracture was his 5.", the period has both roles. We include into the concept of ordinals also certain date formats such as "3.5." (in German, "the third of the fifth"), in opposition to "3.5.2014" (in German, "the third of the fifth, two thousand and fourteen" - and not "fourteenth"). Due to the similarity to the phenomena of abbreviations, we extend the concept of abbreviations to ordinal numbers, arguing that "1." is the abbreviation for "erst(e)(r)" (first), "2." for "zweit(e)(r)" (second) and so on.

The following example from a medical text exhibits numerous short forms, which will be analysed in more detail.

3. St.p. TE eines exulz. sek.knot.SSM (C43.5) li Lab. majus. Level IV, 2,42 mm Tumordurchm.

In "3." the period marks an ordinal number and also a sentence delimiter of the overall short sentence "Thirdly.", introducing an enumerated list item; "St.p." is the abbreviation of "Status post" (state after); "TE" is an acronym derived from "Totale Exzision" (total excision). "Exulz." like "Tumordurchm." are adhoc abbreviations for "exulzerierendes" (fungating) and "Tumordurchmesser" (tumour diameter), respectively. "sek.knot.SSM" is an ill-formed agglutination of two abbreviations and one acronym. In correct writing, they should be separated by spaces ("sek. knot. SSM"). The abbreviation "sek." (secondary) is a common, lexicalized one, whereas "knot." ("knotig", nodular) is, again an ad-hoc creation. "SSM" is an acronym for "Superfiziell Spreitendes Melanom" (superficial spreading melanoma). "C43.5" is a code from the International Classification of Diseases [4]. "Lab." means "Labium", a common anatomical abbreviation. "IV" is not an acronym, but a Roman number. "2,42" is a decimal number, which demonstrates that in German the period is not used as a decimal separator. Finally, the abbreviation "Tumordurchm." demonstrates that the period plays a double role, *viz*. to mark an abbreviation and to conclude a sentence.

## Materials and methods

### Definitions and preprocessing

Based on a preliminary study [5], having applied a unsupervised statistical approach together with a rule-based method for the disambiguation of the period character within clinical narratives, we are focusing in this work on a supervised method exploiting support vector machines for the two different tasks, *viz*. sentence delimitation and abbreviation detection. To this end, we extended a notation introduced by Gillick [6] together with that from Kiss and Strunk [7] to formalize our methodological approach on examples of the form "L*• *R", L*• *representing the left context token, *• *the period character ("."), and R the right context token. Note the token delimiter (here white space) between "L*•*" and "R". From this we derived two tasks:

1. Detection of abbreviations. P(a*|*"L*• *R")

2. Detection of sentence endings. P(s*|*"L*• *R")

A token is the result-output from a tokenizer. We applied the straightforward Lucene [8] based WhiteSpaceTokenizer. As a consequence, periods are always considered part of a token. All new line characters ("*\*n") are preserved before tokenization. As paragraph markers they will be used as features in the classification task. In addition, tokens containing only non-alphanumerical characters were merged with the preceding one. No manual cleansing was performed. Furthermore we introduce *norm*(*L•*) = *L_norm_• *as being a normalization by removing any non-word character except periods. Adjacent periods are merged. *norm*(*R*) = *R_norm _*replaces all non-word characters in *R *getting the *word *content. In our context, we understand (German) abbreviations as being shortened words including a period character at the rightmost position, in contrast to acronyms which never include a period at their rightmost position.

### Data

Our data set was extracted using code-based search across all in- and outpatient discharge summaries from the dermatology department of the Graz University Hospital, covering the period between 01/2007 and 05/2013. The extraction was done using an ETL (Extract Transform Load) workflow with Talend Open Studio [9] and yielded 1,696 summaries. Both extraction and anonymization were mandated by the data owner and conducted by our Scientific Service Area - Medical Data Management group, with the unique purpose to produce a non-identifiable medical corpus for advanced text mining studies. The authors divided the anonymized patient summaries into a training corpus (848 documents) and a test corpus (848 documents).

### Gold standard

We applied the sampling theorem using Chernoff bounds [10] to estimate a statistical representative sample size out of the training and test corpus with the following condition [11]:

(1) $$n\geq \frac{2+\in }{\in^{2}}\text{ln}\frac{2}{\delta }$$

We chose an accuracy of *∈ *= 0.05 and a confidence of 1 *− δ *= 0.95 which corresponds to a sample size of *n *= 3024 text snippets as a representative gold standard size. The advantage of using the estimator theorem is its independence of the overall collection size for estimating a sub sample size. By applying the estimator theorem we claim that a feature estimate or representative syntactical pattern occurrence using the sampled corpus, with a probability of 95% is within +*\−*5% of the truth. Therefore by applying this approach for sub sample size estimation we attempted to fetch a significant amount of linguistic variations, which must be considered for interpreting the period character as an abbreviation or sentence delimiter (or both) and which allows generalizations from the experiments to the whole corpus.

By applying the theorem a reference standard was created through the random selection of 3024 text snippets for both the training and test set, centered on a period followed by a white space or newline, together with its left and right context (each 60 characters) from the sample texts. (For this experiment we did not consider the sporadic cases in which spaces after periods were erroneously omitted). Both authors rated each period character in the center of the snippet as functioning either as an abbreviation marker and/or sentence delimiter. As a measure of inter-rater agreement Cohen's kappa [12,13] was calculated.

### Language resources

Two German word lists were created and indexed: (i) an abbreviation-free medical domain dictionary (MDDict) with a high coverage of domain-specific words, excluding abbreviations, and (ii) a closed-class dictionary (CCDict) containing common, domain-independent word forms.

For **MDDict**, words were harvested from three sources: a free dictionary of contemporary German [14], a word list created out of raw text extracted from an old CD-ROM version of a medical dictionary [15], and medical texts and forum postings from a patient-centered website [16]. All tokens that ended with a period were discarded. The list comprised of about 1.45 million unique word types (the high number is due to inflectional/derivational variants and numerous single-word compounds), which were indexed with Lucene. Due to possible punctuation errors (such as "etc" instead of "etc.") it could not be guaranteed that the dictionary, at this step, was completely devoid of entries that would form a valid abbreviation if appended by a period. This was why it was modified, in a second step, by two web resources containing German abbreviations [17,18]. In total, we accumulated about 5,800 acronym and abbreviation tokens, of which terminal periods were stripped. Matching words were then removed from the initial dictionary.

For **CCDict **we harvested closed-class words from a German web resource [19], i.e. prepositions, determiners, conjunctions, and pronouns, together with auxiliary and modal verbs. The purpose of which was to have a comprehensive list of non-capitalized word forms, the capitalization of which always indicates the initial token of a sentence. The compilation of such a list benefits from a unique characteristic of the German language: namely, that all nouns are capitalized like proper names. Adjectives and full verbs may be capitalized, according to their syntactic role. Therefore, only German closed-class words follow capitalization patterns as in English, which warrants a high coverage for CCDict.

For the harvesting of the afore mentioned web resources we used Apache UIMA [20], for which tailored CollectionReaders were implemented.

### Support vector machines

Support vector machines [21-23] use the following decision function for classifying instance label pairs (**x***_i_, y_i_*), *i *= 1,..., *l *for all **x***_i _*∈ ℝ*^n ^*to a target value *y *∈ {1, −1}:

(2) $$f\left(x\right)=sgn\left(w^{T}\phi \left(x\right)+b\right)$$

(3) $$=sgn\left(\overset{l}{\underset{i=1}{\sum }}\alpha_{i}y_{i}\phi {\left(x_{i}\right)}^{T}\phi \left(x\right)+b\right)$$

(4) $$=sgn\left(\overset{l}{\underset{i=1}{\sum }}\alpha_{i}y_{i}K\left(x_{i},x\right)+b\right)$$

**w **∈ ℝ*^n ^*being a weight coefficient term and *b *∈ ℝ defining a bias. For finding the optimal $\alpha_{i}$ the following minimization problem must be solved [24]:

(5) $$min_{w,b,\xi }=\frac{1}{2}w^{T}w+C\overset{l}{\underset{i=1}{\sum }}\xi_{i}$$

(6) $$subject\;to=y_{i}\left(w^{T}\phi \left(x_{i}\right)+b\right)\geq 1-\xi_{i},\xi_{i}\geq 0$$

*ξ_i _*∈ ℝ defines an upper error bound and *C *∈ ℝ is a tradeoff parameter between the error and margin. Due to the fact that after applying the Lagrangian the final optimization problem depends on the inner product in the form of $x_{i}^{T}x_{j}$ one can use the so-called kernel trick, getting the inner product of a kernel $K(x_{i},x_{j})=\phi {\left(x_{i}\right)}^{T}\phi (x_{j})$ without actually performing the real transformation from the input feature space into a higher dimensional one. The major effect is that instances that are not linearly separable in the input space become linearly separable in the higher dimensional feature space, e.g. the XOR problem [22,25].

Our preference for support vector machines is due to their known good performance on textual data [26] as well as their suitability for binary classification tasks. We were exploiting a linear kernel $K\left(x_{i},x_{j}\right)=x_{i}^{T}x_{j}$ using LIBLINEAR [27] in combination with Weka [28]. In addition we applied the data preprocessing methods described in [29] for generating our instances, especially scaling our features to a range of [-1;1] and normalizing the feature vectors to unit length. The scaling of the test set was dimensioned according to the different value ranges in the training set. For selecting the optimal parameter *C *for the linear kernel we used a meta classifier (CVParameterSelection) varying the parameter *C *on a logarithmic scale [0.001;1000]. According to Joachims [30] "A small value for *C *will increase the number of training errors, while a large C will lead to a behavior similar to that of a hard-margin SVM", therefore not allowing classification errors and having the tendency to over-fit.

We encapsulated Weka and its core methods for training and evaluation into a built-up evaluation framework exploiting UIMA for rapid performance evaluation, getting fast access to true positives, false positives, true negatives and false negatives in the training and test set. Additionally, we implemented the functionality to get the top *n *most relevant features back from the trained model according the method described by Guyon et al. [31], which implies using a linear kernel, as we were interested in feature relevance ranking for both classification tasks. With this information we can state relevance criteria per feature set combination, which are described in more detail in the next two sections.

### Features for abbreviation detection

#### Statistical corpus features

Kiss and Strunk [7] used the *log likelihood ratio *[32] for unsupervised abbreviation detection:

(7) $$log\lambda =-2log\left(P\left(H_{0}\right)/P\left(H_{A}\right)\right)$$

*H*_0 _is the hypothesis that the occurrence of a period is independent of the preceding word, *H_A _*the alternative hypothesis *viz*. that it is not independent. The calculation of *logλ *requires the corpus based frequency counts (Table 1) for every "L*• *R".

**Table 1:** Corpus based frequency counts (C) required for *logλ *calculation.

	*L*	*¬L*
*• *	*C*(*L_norm_, •*)	*C*(*¬L_norm_, •*)
*¬• *	*C*(*L_norm_, ¬•*)	*C*(*¬L_norm_, ¬•*)

According to Dunning [33] one can "restate these counts slightly as the number of times the events occurred together" (in our notation Table 1 *C*(*L_norm_, •*)), "the number of times each has occurred *without *the other" (in our notation Table 1 *C*(*¬L_norm_, •*) and *C*(*L_norm_, ¬•*)) "and the number of times something has been observed that was neither of these events" (in our notation Table 1 *C*(*¬L_norm_, ¬•*)). We exploited the implementation given in the Apache Mahout [34] package for calculating *logλ *as well as building a frequency count map containing the different counts per *C*(*L_norm_, •*) and *C*(*L_norm_, ¬•*) collocation from which we can infer the other two frequency counts. Finally, this feature set comprises *C*(*L_norm_, •*), *C*(*¬L_norm_, •*), *C*(*L_norm_, ¬•*), *C*(*¬L_norm_, ¬•*) and *logλ*.

#### Scaling combinations

As log-likelihood calculation tends to find all abbreviations but generally lacks precision [7], Kiss and Strunk applied different scaling factors to *logλ *for abbreviation [7] and sentence detection [35,36] in combination with a threshold that had been defined by the authors after a series of experiments. In order to avoid setting a threshold arbitrarily, we generated every possible scaling combination of the factors described below and established each unique scaling combination as a separate feature. In combination with feature relevance ranking at the end, after training a model, we can assess the importance of scaling combinations for the classification performance. The following single scaling functions form the base:

(8) $$S_{1}\left(log\lambda ,\;L_{norm}\right):log\lambda \cdot e^{C\left(L_{norm},•\right)/C\left(L_{norm},\neg •\right)}$$

The scaling factor enhances the initial *logλ *if the co-occurrence value *C*(*L_norm_, •*) is greater than *C*(*L_norm_, ¬•*).

(9) $$S_{2}\left(log\lambda ,\;L_{norm}\right):log\lambda \cdot \frac{C\left(L_{norm},•\right)-C\left(L_{norm},\neg •\right)}{C\left(L_{norm},•\right)+C\left(L_{norm},\neg •\right)}$$

This scaling factor *S*_2 _varies from -1 to 1 depending on co-occurrence counts of *C*(*L_norm_, •*) and *C*(*L_norm_, ¬•*). If *C*(*L_norm_, ¬•*) *> C*(*L_norm_, •*) the scaling factor will be negative. If *C*(*L_norm_, ¬•*) *< C*(*L_norm_, •*) the scaling factor will turn positive. The scaling factor equals zero if *C*(*L_norm_, ¬•*) = *C*(*L_norm_, •*).

(10) $$S_{3}\left(log\lambda ,L_{norm}\right):log\lambda \cdot \frac{1}{e^{wordLength(L_{norm})}}$$

This scaling factor punishes long words, based on the observation that most abbreviations are short.

(11) $$S_{4}\left(log\lambda ,L_{norm}\right):log\lambda \cdot \left(\text{number of periods in}\;L_{norm}+1\right)$$

This scaling factor gives an advantage to words that contain an internal period over words having none. The higher the number of internal periods in *word*, the higher is the chance that the word is an abbreviation.

(12) $$S_{5}\left(log\lambda ,L_{norm}\right):log\lambda \cdot \frac{1}{wordLength{\left(L_{norm}\right)}^{C\left(L_{norm},\neg •\right)}}$$

This scaling factor penalizes occurrences of *L_norm _*without a final period exponentially. This means that if they occur frequently, it is less likely to be an abbreviation also with respect to their length.

(13) $$S_{6}\left(log\lambda ,L_{norm}\right):log\lambda +N\left(L_{norm}•\right)$$

We introduced a sixth scaling function *S*_6_, which reflects the fact that most abbreviations are proper substrings of the shortened original word (e.g. "exulz." = "exulzerierend"), with *N *(*L_norm_•*) being the sum of all found substring matches in *L_norm_• *= (*subword*_1 _*• subword*_2 _*• *... *subword_n_•*) for every *subword_i _*in a Lucene search result using MDDict. The reason why the last scaling function contains an addition, is to accommodate for cases where *C*(*L_norm_, •*) *< C*(*L_norm_, ¬•*) even when *L_norm _*is an abbreviation. These cases, for which the weighted *logλ *is negative, could therefore be pushed to the positive side in the result of a strong *S*_6_. The primary *logλ *is modified by sequential composition of all possible variations of scaling factors (calculating the power set *P *(*S*); *S *= {*S*_1_*, S*_2_*, S*_3_*, S*_4_*, S*_5_*, S*_6_}), each resulting combination reflecting a feature.

#### Length features

Like Kiss and Strunk [36] we considered the length of a word as the count of all non-period characters, because internal periods should not have punishing effects.

(14) wordLength(St.p.)=3

For building a descriptive abbreviation length statistic (*µ *= 5.8*, σ *= 4.4) from the training corpus we included those tokens that exhibited a significant *C*(*L_norm_, •*) collocation (*p <*0.01) and *C*(*L_norm_, •*) *> C*(*L_norm_, ¬•*). Using this distribution we formulated the following length dependent features on *L_norm_*:

**Length of candidate**. The absolute length of the abbreviation candidate, counting non-period characters.

**Upper length border**. The values of three right-tailed decision boundaries (*b*_1 _= *µ *+ 1.645*σ, b*_2 _= *µ *+ 1.960*σ, b*_3 _= *µ *+ 2.576*σ*).

**Binary decision rule**. If the candidate is above one of the three different levels (*b*_1_*, b*_2_*, b*_3_). Each decision result is a separate feature.

**Mean minus length**. The mean of the descriptive abbreviation length statistics minus the length of the candidate.

#### Word type features

*L_norm_*, i.e. the word itself forms a feature. In order to keep the feature set small we replaced series of numerical digits with the character "d" within this feature set.

#### Rule-based features

We are exploiting three different binary rules on *L_norm_*:

**Period character inside**. We assumed that the occurrence of at least one additional period character inside the candidate was an important information due to the fact internal periods are suggestive for abbreviations, especially when considering common date formats.

**Contains numerical digit**. This feature is assumed important, as containing digits have a relevant impact on whether the candidate should be classified as non-abbreviation or abbreviation. It is uncommon that tokens which contain digits are abbreviations, with the exception of ordinal numbers.

**All upper case**. Words consisting of upper case characters only are most likely acronyms or common words that were fully capitalized for some reason. The binary information of whether *all *characters within the candidate are upper case is used as a feature.

#### Dictionary-dependent features

This feature requires a dictionary lookup into MDDict, which is assumed to be devoid of abbreviations. If *L_norm _*is found in the dictionary the feature value is set to 1, otherwise to 0.

### Features for sentence detection

#### Abbreviation feature

Whether a candidate is an abbreviation or not constitutes the abbreviation feature. For abbreviation classification we take the best model and its feature combinations from the evaluations described in section *Results of abbreviation detection *and apply it to "L*• *R". If classified as an abbreviation it should favor the decision against sentence delimitation, as most abbreviations tend to appear within and not at the end of a sentence.

#### Length features

We applied the same length features as described in the *Features for abbreviation detection *section as we have seen in our evaluation results for abbreviation classification that this feature set contains two of the top 10 overall features. It is therefore also included into the feature set for sentence detection. The only difference is that here we generate the feature set based on the right context *R_norm_*.

#### Rule-based features

We exploited four different binary rules. The first three were intended to introduce right-context-based abbreviation information to the sentence delimiter decision. The last rule is a direct sentence delimiter rule.

**Contains period**. We assume that the period information with the candidate is an important information due to the fact that most tokens containing an internal period are abbreviations (Applied to *R*).

**Contains digit**. The feature is assumed important, as containing digits have a relevant impact whether the candidate should be classified as a non-abbreviation or abbreviation (Applied to *R_norm_*).

**All upper case**. Capitalized words are acronyms or emphasized words. If followed by a period they should be classified as non-abbreviations (Applied to *R_norm_*).

**Capitalization**. The capitalization of the first character of *R_norm _*is a good indicator for sentence delimiters, because new sentences generally begin with an upper case character.

#### Word type features

This feature set was generated in the same way as described in the *Features for abbreviation detection *section, i.e using word type information. We also included this information for sentence detection, because previous tests on abbreviation detection had shown that this feature set, in isolation, had produced an unweighted micro-averaged F-measure of 0.93 for a 10-fold cross validation on the training dataset (Table 2). High performance values for sentence delineation with this feature set alone has also been reported by Gillick [6].

#### Right context word type features

This feature set was generated in the same way as described in the previous section, the only difference being that here it was applied to the right context *R_norm_*. We decided against using an n-gram representation as this still allows the exploitation of some right context type information *R_norm_*, in the case that the type information of *L_norm _*is missing, or vice versa. The possibility for using *R_norm _*and *L_norm _*as a combined feature set, has the advantage that it is not as strict as a stand alone bi-gram (*L_norm_, R_norm_*) feature set. However, the bi-gram information is encoded if existent and the feature set is kept small.

#### Text formatting features

In well-formatted text a new line character after a period marks the end of a paragraph. Therefore, the period here can generally be assumed to play the role of a sentence delimiter, because sentences never span across paragraphs. In our case we could not strictly rely on this rule, as parts of the clinical narratives under scrutiny were fragmented with new line characters "*\*n". This was the result of a side effect stemming from the process that extracted the narratives out of the clinical information system, a phenomenon which is also well known when extracting raw text from PDF sources. As a consequence, only double new line characters could be safely considered as paragraph markers. Nevertheless we wanted to investigate the impact of this feature on the sentence detection task. We therefore formulated the following features:

**Single new line**. The feature is set true if *R *starts with a single new line.

**Double new line**. The feature is set true if *R *starts with a double new line.

**No new line**. The feature is set true if *R *starts with no new line.

#### Language-dependent features

Similarly to the *Features for abbreviation detection *section we performed a lexicon lookup of *R_norm _*in MDDict in order to decide whether *R_norm _*existed in the harvested dictionary. We hypothesized that this was also important for sentence detection because it seemed that a sequence of two abbreviations normally occurred within the same sentence.

## Results

We report on our evaluation results, starting with 10-fold cross validation on the training set. Afterwards, the trained model was used, for the performance evaluation of the test set. Results are provided as unweighted micro-averaged F-measures, as recommended by Manning et al. [37].

A Cohen's kappa of 0.98 clearly reflects the fact that both abbreviation and sentence detection are easy tasks for human raters. By identifying the top 10 relevant features depending on the formed feature sets from the trained model, we could state the impact of these features on the classification tasks. We applied significance tests (chi-squared test, *p <*0.05) with respect to the base line and on different ranked feature set combinations.

### Results of abbreviation detection

As a baseline we chose the following straightforward decision algorithm: if the abbreviation candidate is followed by a lower case character it is classified as abbreviation, otherwise as non-abbreviation.

First we evaluated the feature sets for abbreviation detection in isolation, of which the achieved performance values are depicted in Table 2. We are referring to the training set in the following exemplification. The rule-based features showed poorer performance, also significantly inferior to the very straightforward baseline. Nevertheless, within this feature set (Table 3), the feature *Contains period *appears as the most relevant feature for this task. Following the rule-based features, the statistical feature set has, in isolation, a micro-averaged F-measure of 0.73 with the simple frequency count *C*(*L_norm_, •*), listed as the most relevant within this feature set. After the rule-based feature set, the scaling, language-dependent and length feature sets achieve, in isolation, the same performance of 0.83. In this setting, interestingly, calculation-intensive statistical feature sets (scaling feature set, length feature set) result in roughly the same performance as a simple dictionary lookup. A respectable performance is achieved using only word type features (*L_norm_*) yielding an F-measure of 0.93. The top 10 features within this set reflect the most common abbreviations within the corpus under scrutiny (Table 3). Only slightly lower performance values were achieved for the test set.

**Table 2:** Abbreviation detection.

Method	BL	1	2	3	4	5	6
micro-avg. F_1 *Training*_	0.62	0.60*'	0.73*'	0.83*'	0.83*	0.83*	0.93*'
micro-avg. F_1 *Test*_	0.60	0.60	0.70*'	0.81*'	0.83*	0.84*'	0.92*'

Evaluation performance per feature set (1 Rule-based features; 2 Statistical features; 3 Scaling features; 4 Language-dependent features; 5 Length features; 6 Word type features). ^* ^significant difference to base line (BL) (*p <*0.05), ' significant difference to predecessor (*p <*0.05)

**Table 3:** Abbreviation detection.

Top 10	1	*w* ^2^	2	*w* ^2^	3	*w* ^2^
1	Contains period	0.30	*C*(*L_norm_, •*)	1.34	*S*2	3897.48
2	All upper case	0.02	*logλ*	0.80	*S*3	3222.35
3	Contains digit	0.01	*C*(*L_norm_, ¬•*)	0.43	*S*4	2592.76
4	-	-	*C*(*¬L_norm_, ¬•*)	0.31	*S*2*, S*3	2329.77
5	-	-	*C*(*¬L_norm_, •*)	0.19	*S*4*, S*5	847.88
6	-	-	-	-	*S*5	706.98
7	-	-	-	-	*S*2*, S*4*, S*5	511.38
8	-	-	-	-	*S*2*, S*5	412.86
9	-	-	-	-	*S*3*, S*4	204.80
10	-	-	-	-	*S*2*, S*3*, S*4	139.36

**Top 10**	**4**	***w* ^2^**	**5**	***w* ^2^**	**6**	***w* ^2^**

1	∈ MDDict	0.34	LT border *b*_2_	16.15	St.p.	409.58
2	-	-	LT border *b*_1_	16.15	Amb.	409.51
3	-	-	LT border *b*_3_	16.15	o.B.	409.09
4	-	-	LT	8.74	re.	407.87
5	-	-	Mean-LT	8.74	Z.n.	407.35
6	-	-	*> b*_1_	0.54	li.	407.28
7	-	-	*> b*_3_	0.16	ca.	407.00
8	-	-	*> b*_2_	0.10	unauff.	406.94
9	-	-	-	-	bds.	406.19
10	-	-	-	-	Pat.	405.75

Top 10 feature rankings per feature set (1 Rule-based features; 2 Statistical features; 3 Scaling features; 4 Language-dependent features; 5 Length features; 6 Word type features). Length (LT); *w*^2^: Weight based feature relevance criterion.

After the evaluation of each feature set in isolation we combined these sets step wise and evaluated their combined performance (Table 4). We started with the rule-based feature set and added the statistical feature set, achieving an F-measure of 0.71. By combining these two feature sets we obtained a lower performance than when using only the statistical set in isolation, comparing Table 2 with Table 4 for the training set. In the next step we added the scaling features, yielding in combination, an F-measure of 0.86 which is higher compared to the scaling features in isolation. Interestingly, when analysing this combined set (Table 5), the top 10 features were constituted by only scaling combinations and *logλ*. The same was true when adding the language-dependent set, with only the ranking being different. Nevertheless when adding the language-dependent feature set we obtained a performance gain up to an F-measure of 0.88 for the training set, thus achieving a positive combined impact. After introducing in the length features set, we obtained an F-measure of 0.95, and, finally together with the word type features, we achieved the highest performance of 0.97 for the training set (Table 4). The final top 10 features of this set are shown in Table 5. It is remarkable that within this ranking *no language-dependent features exist*, but at least one feature belonging to the other feature sets. An F-measure of 0.95 is achieved on the test set by combining all features.

**Table 4:** Abbreviation detection.

Method	BL	[1]	[1-2]	[1-3]	[1-4]	[1-5]	[1-6]
micro-avg. F_1 *Training*_	0.62	0.60*'	0.71*'	0.86*'	0.88*'	0.95*'	0.97*'
micro-avg. F_1 *Test*_	0.60	0.60	0.71*'	0.83*'	0.86*	0.93*	0.95*'

Evaluation performance combining feature sets stepwise according to their stand alone performance (1 Rule-based features; 2 Statistical features; 3 Scaling features; 4 Language-dependent features; 5 Length features; 6 Word type features). ^* ^significant difference to base line (BL) (*p <*0.05), ' significant difference to predecessor (*p <*0.05)

**Table 5:** Abbreviation detection

Top 10	[1]	*w* ^2^	[1-2]	*w* ^2^	[1-3]	*w* ^2^
1	Contains period	0.30	Contains period	0.35	*S*_2_	5885.83
2	All upper case	0.02	*C*(*L_norm_, •*)	0.18	*S*_3_	4855.66
3	Contains digit	0.01	*logλ*	0.13	*S*_4_	1999.51
4	-	-	*C*(*¬L_norm_, ¬•*)	0.12	*S*_2_*, S*_3_	1798.60
5	-	-	*C*(*¬L_norm_, •*)	0.09	*logλ*	1180.39
6	-	-	*C*(*L_norm_, ¬•*)	0.09	*S*_5_	894.98
7	-	-	All upper case	0.02	*S*_4_*, S*_5_	715.70
8	-	-	Contains digit	8.16E-5	*S*_2_*, S*_5_	617.98
9	-	-	-	-	*S*_2_*, S*_4_*, S*_5_	474.86
10	-	-	-	-	*S*_3_*, S*_4_*, S*_5_	256.81

**Top 10**	**[1-4]**	***w* ^2^**	**[1-5]**	***w* ^2^**	**[1-6]**	***w* ^2^**

1	*S*_2_	1063.78	*S*_5_	1027.15	LT	952.62
2	*S*_3_	962.33	*S*_4_*, S*_5_	914.02	Mean-LT	952.62
3	*S*_2_*, S*_3_	507.82	*S*_2_*, S*_5_	610.69	All upper case	549.64
4	*S*_4_	391.68	*S*_2_*, S*_4_*, S*_5_	527.28	*S*_3_*, S*_4_*, S*_5_	529.85
5	*S*_3_*, S*_4_*, S*_5_	379.70	*S*_2_	463.94	*S*_3_*, S*_5_	521.60
6	*S*_3_*, S*_5_	325.68	*S*_3_*, S*_4_*, S*_5_	274.81	erforderl.	403.54
7	*S*_5_	265.62	*S*_3_*, S*_5_	253.30	pathol.	392.23
8	*S*_4_*, S*_5_	222.55	Mean-LT	145.91	verschiebl.	375.40
9	*logλ*	143.67	LT	145.91	d-lat.	358.11
10	*S*_2_*, S*_5_	129.90	*S*_2_*, S*_4_	90.13	entzündl.	345.21

Top 10 feature rankings per feature set (1 Rule-based features; 2 Statistical features; 3 Scaling features; 4 Language-dependent features; 5 Length features; 6 Word type features). Length (LT); *w*^2^: Weight based feature relevance criterion.

### Results of sentence detection

The baseline algorithm for sentence detection analyses the capitalization status of *R_norm_*. Only if capitalized, "L*• *R" is classified as sentence delimiter.

As depicted in Table 6 the language features and the rule-based features alone performed significantly worse than the baseline. Interestingly, using the text format features, even though the texts under scrutiny were heavily contaminated by new line characters, we obtained a performance above the baseline for the first time. The feature relevance ranking at this stage is shown in Table 7. There was no significant performance difference using the word length features in isolation compared to the text formatting features for the training set. The right context word type feature set based on *R_norm _*performed worse than the word type based features using *L_norm_*. The most important features in this set are shown in Table 8. We observed the best performance for the standalone feature set evaluation using only the information specifying whether "L*• *R" is an abbreviation or not, using the optimized SVM for abbreviation detection resulting in an F-measure of 0.92 for sentence delineation. We obtained the same performance on the test set. This reflects the important influence of abbreviation detection on sentence delineation.

**Table 6:** Sentence detection.

Method	BL	1	2	3	4	5	6	7
micro-avg. F_1 *Training*_	0.78	0.58*'	0.76*'	0.79*'	0.79*	0.82*'	0.90*'	0.92*'
micro-avg. F_1 *Test*_	0.75	0.60*'	0.74*'	0.78*'	0.81*'	0.77*'	0.87*'	0.92*'

Evaluation performance per feature set (1 Language features; 2 Rule-based features; 3 Text format features; 4 Word length features; 5 Right context word type features; 6 Word type features; 7 Abbreviation feature). ^* ^significant difference to base line (BL) (*p <*0.05), ' significant difference to predecessor (*p <*0.05)

**Table 7:** Sentence detection

Top 10	1	*w* ^2^	2	*w* ^2^	3	*w* ^2^
1	∈ CCDict	0.07	Capitalization	1.84	No "*\*n"	0.32
2	∈ MDDict	2.15E-3	All upper case	0.54	Double "*\*n"	0.06
3	-	-	Contains digit	0.27	Single "*\*n"	0.03
4	-	-	Contains period	1.59E-5	-	-
5-10	-	-	-	-	-	-

Top 10 feature rankings per feature set (1 Language features; 2 Rule-based features; 3 Text format features). *w*^2^: Weight based feature relevance criterion.

**Table 8:** Sentence detection.

Top 10	4	*w* ^2^	5	*w* ^2^	6	*w* ^2^	7	*w* ^2^
1	LT border *b*_2_	60.82	Die	121.98	St.p.	415.20	Abbr	0.54
2	LT border *b*_1_	60.82	für	121.03	Amb.	410.82	-	-
3	LT border *b*_3_	60.82	TE	94.84	ca.	402.62	-	-
4	LT	1.98	Keine	94.09	Pat.	401.16	-	-
5	Mean-LT	1.98	Sono	83.67	max.	397.93	-	-
6	*> b*_2_	0.13	Der	80.47	Z.n.	392.47	-	-
7	*> b*_3_	0.04	CT	77.13	st.p.	390.62	-	-
8	*> b*_1_	2.27E-4	E-Nr	75.40	n.	378.70	-	-
9	-	-	Im	71.92	St.	377.24	-	-
10	-	-	Am	66.45	bzw.	368.27	-	-

Top 10 feature rankings per feature set (4 Word length features; 5 Right context word type features; 6 Word type features; 7 Abbreviation feature). Length (LT); *w*^2^: Weight based feature relevance criterion.

As in the *Results of abbreviation detection *section we performed a stepwise combination of feature sets in order to gain insight into their combined performance. We achieved the first positive significant impact on classification performance, in comparison to the stand alone evaluation, when combining the first three feature sets, reaching an F-measure of 0.88 for the training set (Table 9). The feature relevance ranking at this point is depicted in Table 10. Adding stepwise word length features, right context word type features (*R_norm_*), word type features (*L_norm_*), and, finally, the abbreviation information we obtained an unweighted micro-averaged F-measure of 0.97 for sentence detection using the training set and an F-measure of 0.94 for the test set (Table 9). Table 11 documents interesting insights into the most relevant features of the top performing model. The information whether "L*• *R" is an abbreviation or not is the most important one, followed by upper case information relating to *R_norm_*. The remaining top 10 features are a combination of word type features (right and left context) and text format features. It is plausible that the text format features convey important information, but it has to be emphasized that the single occurrence of a new line is not in the top features anymore. Due to the contamination of our text sample by new line characters, this feature does no longer reliably predict sentence boundaries. This information has been automatically induced from the parameter and the feature-optimized SVM. The remaining important text format features for sentence detection are Double "*\*n", marking and end of a text passage, and No "*\*n".

**Table 9:** Sentence detection.

Method	BL	[1]	[1-2]	[1-3]	[1-4]	[1-5]	[1-6]	[1-7]
micro-avg. F_1 *Training*_	0.78	0.58*'	0.76*'	0.88*'	0.92*'	0.95*'	0.96*'	0.97*'
micro-avg. F_1 *Test*_	0.75	0.60*'	0.75'	0.86*'	0.91*'	0.93*'	0.94*'	0.94*

Evaluation performance combining feature sets stepwise according to their stand alone performance (1 Language features; 2 Rule-based features; 3 Text format features; 4 Word length features; 5 Right context word type features; 6 Word type features; 7 Abbreviation feature). * significant difference to base line (*p <*0.05), ' significant difference to predecessor (*p <*0.05)

**Table 10:** Sentence detection

Top 10	[1]	*w* ^2^	[1-2]	*w* ^2^	[1-3]	*w* ^2^
1	∈ CCDict	0.07	Capitalization	2.67	Capitalization	1.54
2	∈ MDDict	2.15E-3	All upper case	0.47	No "*\*n"	1.09
3	-	-	∈ CCDict	0.43	∈ CCDict	0.58
4	-	-	Contains digit	0.21	Double "*\*n"	0.48
5	-	-	Contains period	0.02	All upper case	0.17
6	-	-	∈ MDDict	8.32E-4	Single "*\*n"	0.11
7	-	-	-	-	Contains digit	0.07
8	-	-	-	-	∈ MDDict	0.03
9	-	-	-	-	Contains period	0.01
10	-	-	-	-	-	-

Top 10 feature rankings per feature set (1 Language features; 2 Rule-based features; 3 Text format features). *w*^2^: Weight based feature relevance criterion.

**Table 11:** Sentence detection.

Top 10	[1-4]	*w* ^2^	[1-5]	*w* ^2^
1	Capitalization	11.34	LT	674.21
2	LT	10.52	Mean-LT	674.21
3	Mean-LT	10.52	Capitalization	637.85
4	No "*\*n"	4.82	Rippenanteile*_RC_*	627.54
5	∈ CCDict	4.08	Lymphknoten*_RC_*	356.25
6	Double "*\*n"	3.77	Double "*\*n"	336.64
7	All upper case	0.97	Lungengerüstzeichnung*_RC_*	332.50
8	Contains digit	0.71	Integument*_RC_*	321.86
9	*> b*3	0.31	No "*\*n"	300.18
10	Contains period	0.19	Normale*_RC_*	277.68

**Top 10**	**[1-6]**	***w* ^2^**	**[1-7]**	***w* ^2^**

1	Capitalization	971.25	Abbreviation	1326.41
2	Mean-LT	840.45	Capitalization	867.06
3	LT	840.45	o.B.	382.83
4	Double "*\*n"	341.46	Double "*\*n"	374.57
5	No "*\*n"	324.25	No "*\*n"	364.32
6	o.B.	259.13	bds.	282.13
7	Rippenanteile*_RC_*	254.91	CT*_RC_*	266.54
8	mitresez.	254.91	Leberlappen*_RC_*	225.08
9	CT*_RC_*	251.41	A.	206.77
10	Leberlappen*_RC_*	236.26	Narbige*_RC_*	191.01

Top 10 feature rankings per feature set (1 Language features; 2 Rule-based features; 3 Text format features; 4 Word length features; 5 Right context word type features (RC); 6 Word type features; 7 Abbreviation feature). Length (LT); *w*^2^: Weight based feature relevance criterion.

## Related work

The detection of abbreviations and, which is closely related, the identification of the syntactic function of punctuation characters is important due to the frequency of both phenomena in clinical narratives [3]. There are several previous works on the disambiguation [38,39] and normalization of short forms, with the goal to resolve the correct long form depending on its context. CLEF 2013 [40] started a task for acronym/abbreviation normalization, with a focus on mapping acronyms and abbreviations to concepts in the Unified Medical Language System (UMLS) [41]. An F-measure of 0.89 was reported by Patrick et al. [42].

Xu et al. [1] tested four different abbreviation detection methods. The first one formed the baseline. Any unknown token within the narrative in comparison to a medical term list from MedLEE [43,44] (containing 9,721 types plus Knuth's list of 110,573 American English words) was considered an abbreviation. The second was a rule-based approach that was customized by observing different admission notes, e.g. detecting whether the token contains a "." or "-" character. The third method used decision tree classifiers, using features of word formation and frequency, while the fourth method used additional features from other knowledge resources. Six admission noted formed the training set, four were used as a test set. The fourth method performed best with a precision of 91.4% and a recall of 80.3%.

Due to the good performance measure Wu et al. [45] compared three machine learning methods (Decision Tree, Support Vector Machine, Random Forest) for abbreviation detection. The training set comprised of 40 discharge summaries annotated by three experts; another 30 documents constituted the test set. Five different categories made up the full feature space: word formation, vowel combinations, related content from knowledge bases, word frequency in the overall corpus and local context. The Random Forest classifier performed best with an F-measure of 94.8% (precision 98.8%, recall 91.2%). A combination of classifiers lead to the highest F-measure of 95.7%. In addition, Wu et al. [46] compared different clinical NLP systems on handling abbreviations in discharge summaries. MedLEE performed best with an F-score of 0.601 for all abbreviations. The implemented system, which addresses real-time constraints, is described in [47].

Our work is only partially comparable, because it combines sentence delimitation with abbreviation detection. It is also peculiar due to the fact that the period character is mandatory as a non-acronym abbreviation marker in German, which causes severe disambiguation problems. In contrast to other work, we refrained from investigating acronyms. Is is perhaps true that our notion of an abbreviation is idiosyncratic, compared to the more general meaning of the term, especially regarding the English language, where abbreviations are often defined as any type of shortened term, including acronyms ("MI - Myocardial Infarction"), shortened words or phrases (e.g., "pt - patient"), and symbols (e.g., "eth - ethanol") [45]. Nevertheless, our distinction seems justifiable in the light of the particularities of German language, especially medical sub-language, for which - to the best of our knowledge - this investigation constitutes the first study on sentence delineation with the additional focus on abbreviation detection. In a preliminary study [5] we addressed the problem of sentence boundary detection together with abbreviation detection on similar texts. We had combined an unsupervised statistical approach together with a rule-based method for the disambiguation of the period character. As a result we obtained an accuracy of 0.93 for sentence boundary detection and abbreviation detection. Cases in which the periods were preceded by numerical characters were excluded in that study, therefore the results are not fully comparable.

## Conclusion and outlook

We have presented and evaluated a supervised machine learning approach using a support vector machine exploiting a linear kernel for two tasks, namely abbreviation and sentence delineation in German-language medical narratives. The technical implementation was based on the UIMA framework in conjunction with Weka. We created a modular evaluation framework to gain insight in different classification settings and feature relevance rankings. Exploiting this framework we managed to achieve, for abbreviation detection, an unweighted micro-averaged F-measure of 0.97 for the training set and an F-measure of 0.95 for test set based evaluation. For sentence boundary detection we report an unweighted micro-averaged F-measure of 0.97 for training set based evaluation and an F-measure of 0.94 using the test set. We achieved a comparable performance to the maximum entropy based sentence detection tool implemented within OpenNLP [48], exploited by cTakes [49] (sentence boundary detector accuracy = 0.949). Both results are remarkable as clinical narratives have specific idiosyncrasies (telegram-style, numerous ad-hoc abbreviations, spelling and typing errors), and are thus quite distinct from the proof-read content of textbooks and scientific papers [50].

Future work may explore how the achieved performance for abbreviation detection and sentence delineation can be enhanced by exploring additional feature sets, in order to reduce the final false positive and false negative rate. This could be done by exploiting n-gram information, expanded context information, additional corpus-based statistical features, or word formation features as described by Wu et al. [45]. Due to the fact that we achieved comparable results to the cTakes sentence detection tool (applied to English clinical text) using OpenNLP, a direct comparison between the approach presented in this paper, and a retrained version of the OpenNLP sentence detection tool for our German texts would be interesting for a supervised approach in general. Additionally, an enhanced version of the preliminary approach described in [5] could be further evaluated. Furthermore, the applicability to other clinical subdomains would be of interest, as different document types (e.g. Dermatology Clinic Notes, Neurology Clinic Notes) form distinct sublanguages, according to Friedman [51] and Patterson et al. [52]. Interinstitutional and interregional evaluations (e.g. comparing Austria, Germany, Switzerland for the German speaking community) could be investigated, in order to obtain more generally applicable NLP pipelines for medical document processing and to identify the needs for customization. Further work may also propose additional features that are language-independent and do not rely on language-specific dictionaries or rules. Language-independent implementations, also considering real-time constraints in a clinical setting, could further improve current clinical NLP frameworks, such as cTakes or MedKAT [53] for the non-English clinical NLP community.

## Competing interests

The authors declare that they have no competing interests.

## Authors' contributions

Markus Kreuzthaler performed the technical implementation and the experiments. Both authors contributed to the methodology, the selection of the features, and annotated test and training data.

## Grants

Publication charges for this work was funded by the European Union's Seventh Framework Programme for research, technological development and demonstration under grant agreement n*^o ^*611388 - the SEMCARE project.
